# A Design Process Framework and Tools for Teaching and Practicing Biomimicry

**DOI:** 10.3390/biomimetics10060376

**Published:** 2025-06-06

**Authors:** Benjamin Linder, Jean Huang

**Affiliations:** Olin College, Needham, MA 02492, USA; jhuang@olin.edu

**Keywords:** abstraction, analogy, biomimicry, framework, design process, methodology

## Abstract

Few design methods exist that provide clearly structured, visually intuitive, and easily monitored scaffolding for navigating the considerable complexity of biomimetic processes. To this end, we present a holistic biomimicry process framework informed by design abstraction models that clarifies core skills involved and how they combine to form essential practices, such as biology-to-design and challenge-to-biology. The structure–function and conditions-conducive-to-life perspectives are facilitated, ensuring support for sustainability considerations. We pair this framework with two process tools that make biomimicry design moves explicit for learners, educators, and practitioners. We share examples of the use of these tools from an upper-level undergraduate engineering course in biomimicry. Our observations indicate that the framework and tools support process acquisition, design iteration, knowledge transfer, and sustainability integration in biomimicry education and practice by enabling common design behaviors such as variation, iteration, debugging, and reflection.

## 1. Introduction

Biomimicry, or biologically inspired design (BID), draws on strategies and mechanisms from biological systems for insight into human design challenges. Despite its potential to support sustainable innovation, BID remains underutilized in practice due to methodological gaps, difficulties in knowledge transfer, and challenges in interdisciplinary collaboration [1,2]. We present a structured, visually intuitive scaffolding to support practitioners in systematically navigating the biomimetic design process.

The current work introduces a biomimicry process framework that synthesizes BID practices and sustainability considerations. By structuring biomimicry process as a sequence of design moves—involving abstraction and analogy in the form of Observation, Translation, and Application—the framework can provide clarity and facilitate usability for novice learners and experienced practitioners.

A challenge in BID is the effective transfer of biological insights into applications, and studies have highlighted the need for better ways to facilitate abstraction and translation between disciplines [1,2]. To address this, we pair a process framework with tools for explicitly tracking design decisions, supporting common behaviors such as iteration, variation, reflection, and debugging. This approach aligns with recent recommendations for improving BID’s accessibility and implementation [1]. Furthermore, while BID has been recognized for its potential to support sustainability, this aspect is not always explicitly integrated into design frameworks. The framework presented can support consideration of sustainability through the biomimetic design process when combined with pedagogy that highlights perspectives from structure–function and conditions-conducive-to-life aspects of behavior of natural systems [3]. The integration supports BID as a mechanism for promoting holistic sustainable design insights.

By presenting a structured framework and corresponding tools for implementing interdisciplinary collaboration and knowledge transfer, including sustainability considerations, we provide practical support for enabling holistic biomimicry practice. This work contributes to the growing field of BID by providing a systematic, scalable, and accessible methodology adaptable for educators, learners, and practitioners.

## 2. Motivation

A biomimetic process involves a considerable amount of information and complexity (depending only somewhat on how it is practiced) [4], especially one that attempts to learn structure–function insights integrated with conditions-conducive-to-life ones to realize full sustainability [3]. One full biomimicry design process cycle (challenge-to-biology) involves many steps that are not obvious to the uninitiated. Furthermore, the overall result is a product of each of these steps over the cycle, so early design choices have an outsized influence as the process progresses. Thus, new practitioners can be anxious and unsure even when they are achieving satisfactory results, asking “Is this right?” and “Is this what I should be doing?” We sought a way to communicate and manage a fuller biomimicry process, prioritizing accessibility for new learners and practitioners.

Without a clear map of the process, new learners and practitioners can find it difficult to understand where design outcomes come from, have confidence in those outcomes, adapt the process to different needs, or retrace their steps to diagnose and refine or debug the outcomes. These abilities are particularly important because there are many common errors or misconceptions that can occur in biomimicry practice [4,5,6]. And the aspects of variation and iteration that are so important in design practice can be difficult to manage if the underlying design process is not stable and interrogatable.

We, as a designer and biologist teaching engineering students, sought a process framework that could provide clearly structured, visually intuitive, and easily monitored scaffolding to aid practitioners in navigating and keeping track of the steps and pathways in one full cycle of a biomimicry process. To increase accessibility, we leveraged readily available terminology, materials, and practices.

Existing BID frameworks can visually emphasize the iterative nature of the process, making multiple design cycles explicit [7,8,9,10,11]. However, we found less experienced learners wanting more direction on each of the steps and their interrelationships within a design cycle. Existing frameworks emphasize problem-solving as the primary purpose and nature of the process [7], often including elements that are not specific to biomimicry, e.g., problem definition and evaluation, and de-emphasizing biomimicry elements, e.g., the amount of abstraction and specification involved. We chose to emphasize the primary design moves of analogy and abstraction and de-emphasize problem-solving to present a clearer, more universal conceptual picture of the practice.

We did not explore how our framework would be adapted for specific application areas, which have proliferated [7]. While we provide instruction to our students on performing the design moves involved, we believe more attention to expert versus novice experience is needed for advancement [2,12]. For example, we potentially see evidence of design fixation by students trained in this way [13,14].

## 3. Materials and Methods

### 3.1. Visual Process Framework

We took a constructive approach to the creation of a visual framework, by looking at the BID process from the perspective of people attempting to take inspiration from Nature to address a design challenge. We were simultaneously informed by our familiarity with the biomimicry repertoire as it is commonly taught and practiced in workshops, classes, and programs we experience in the U.S. context.

**Figure 1:**
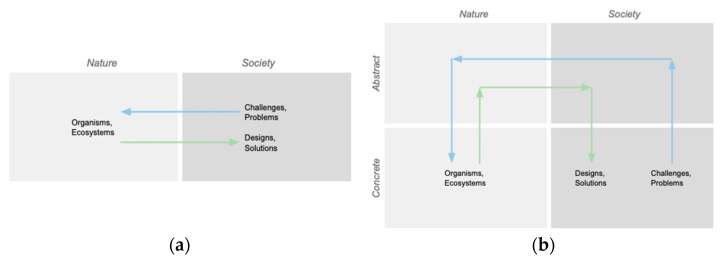
(**a**) The basic design-by-analogy moves from the Society domain to the Nature domain (blue arrow) and back (green arrow) that characterize biomimicry processes. (**b**) The design abstraction moves necessary to practically carry out the design-by-analogy moves.

At its most basic, the process involves taking a design challenge and looking to Nature for insight into the challenge (Figure 1a) [15], which is to say, the practice involves translating from the Societal domain to the Nature domain and back again (or directly from Nature to society in a solution-based exploration [16]) through analogy. Biomimicry’s status as a design-by-analogy (DbA) process is well known [17,18,19,20,21], and biomimicry examples are often cited when describing the use of analogy in design [22].

In practical terms, the real-world context of interest is too richly complex and detailed or unknown to be translated directly to the Nature domain, necessitating selection and simplification through abstraction. The same is true for the particulars of the natural world that must be reduced before translating back to the Societal domain. The repeated steps of abstraction and specification (de-abstraction) of one design cycle are shown in the 2 × 2 framework in Figure 1b. Our recognition of this role of abstraction was informed by design abstraction models of the design process more broadly [23,24]. See Graeff et al. for an analysis of abstraction in the context of BID [2].

These observations led us to see biomimicry as a design process fundamentally involving analogy and abstraction as the primary design moves. These happen to be some of the most powerful moves in design, illuminating for us the general power of biomimicry. We imagine that experienced practitioners are tacitly able to navigate these moves without necessarily realizing all that they are doing in this regard [25].

**Figure 2:**
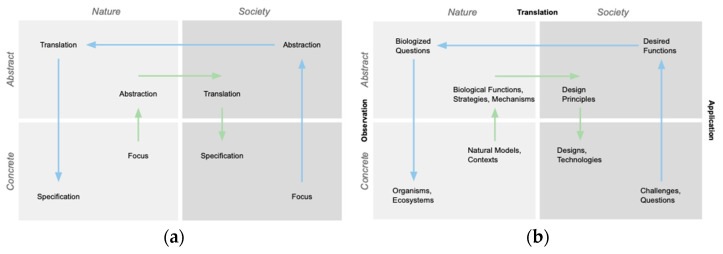
(**a**) A framework for understanding the general abstraction and analogy steps of a biomimicry process. (**b**) The framework with common biomimicry design terms mapped to the steps.

This framing simplifies the full process into a set of eight primary steps, many of which are duals of each other (Figure 2a). These steps are (1) the selection of the focus in the Societal domain, (2) abstraction in the Societal domain, (3) translation to the Nature domain, (4) specification in the Nature domain, (5) selection of the focus in the Nature domain, (6) abstraction in the Nature domain, (7) translation to the Societal domain, and (8) specification in the Societal domain. While these steps are normative, they are intended to be dynamically and contingently deployed through feedback, variation, and iteration, including nonlinearly by more experienced practitioners [5].

We found these steps correspond well with common biomimicry design practices, and a mapping of selected terms [5,17,26] describing the outcomes or design representations resulting from each of these steps is provided in Figure 2b. Although developed independently, these steps also correspond well with eight-step models identified by others [2,11]. These steps de-emphasize problem-solving elements that are common to many frameworks. See Fayemi et al. for a comparison of the steps in different biomimetic process models [11].

These terms are not unique, and this choice can be consequential [2], especially without the support of clarifying instruction. This generalized design process can also be carried out by changing the constructs or design representations involved in each step. For example, while abstracting functions from a challenge is common in biomimicry practice, especially in the engineering context like ours [27], this choice could be replaced with another aspect, such as aesthetics.

There are also common terms for types of BID design moves in different contexts [11,26], and we mapped Application (A) as describing the abstraction and specification moves in the Societal domain, Translation (T) as describing the analogy moves between the domains, and Observation (O) as describing the abstraction and specification moves in the Societal domain (Figure 2b). Translation moves between domains are often referred to as biologizing and de-biologizing depending on the direction of translation. We have taken to referring to these classes of moves as skills to be practiced [16,26]. Fayemi et al. use Analysis, Abstraction, Transfer, and Application [11].

**Figure 4:**
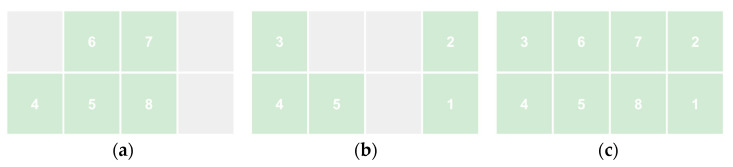
(**a**) Portions of the framework template used for the biomimicry processes biology-to-design, (**b**) challenge-to-biology, and (**c**) challenge-to-biology-to-design with the corresponding steps involved numbered and highlighted in green.

This framework gives us a common language for speaking about different design moves and sets of moves to support conversations with students and practitioners about their design activities. For example, a biology-to-design (solution-based or technology push) practice [16,17,28,29] can be expressed in shorthand as OTA, combining the O, T, and A design moves into a design process (Figure 4a). A challenge-to-biology (problem-driven or technology pull) practice is represented in shorthand by ATOTA (Figure 4b). Other processes are possible; for example, we have used an ATOT process to identify design principles for sustainability [3]. We found this ability to express sets of moves in shorthand useful for encapsulating complexity, allowing us to shift fluidly between the general and the particular with students and practitioners. We have applied this framework in both our classroom teaching and industry consulting work over many years.

### 3.2. Design Process Tools

**Figure 3:**
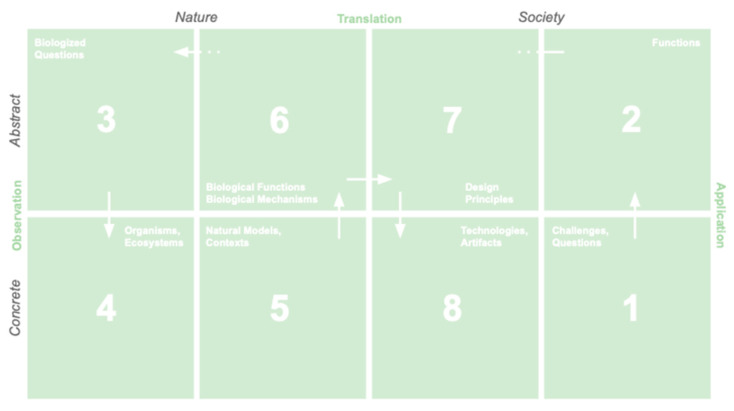
A process template to organize and guide a biomimicry design process. Each of the steps in a full challenge-to-biology (to design) process are numbered 1–8 with an arrow at each step pointing to the next step.

We developed two design tools for scaffolding the framework in education and practice settings. The first is a process template of the framework that maintains the visual organization, includes the terminology for guidance, and provides space to record the results of each step (Figure 3). Users navigate the template by completing the boxes associated with the steps for their chosen process (Figure 4), with the arrows between the steps providing hints for the sequencing to follow. The outputs of the steps are hinted with labels in the boxes, e.g., the output of step 3 is biologized questions. Steps 1, 2, 7, and 8 are indicated as being in the Society domain, and steps 3, 4, 5, and 6 are seen to be in the Nature domain. Steps 2, 3, 6, and 7 are indicated as involving abstract concepts, whereas steps 1, 4, 5, and 8 involve concrete, in-the-world elements. The adjacency of the steps is informative, e.g., the design principles resulting from step 7 are the insights that respond to the functions sought in step 2 next to it.

We have found the template is particularly useful for learning and understanding the process structure, the types of design moves, and the outputs involved. The template visually emphasizes the steps and details of one design cycle and de-emphasizes the cyclic nature of the process often depicted in frameworks [7,8,9,10,11]. The template can be thought of as a biomimicry canvas in that it provides an organized layout of all of the steps on one sheet [30].

The template can be used for a range of biomimicry processes that share these steps, which include biology-to-design, challenge-to-biology, and challenge-to-biology-to-design (often shortened to challenge-to-biology), as shown in Figure 4a–c.

**Figure 5:**
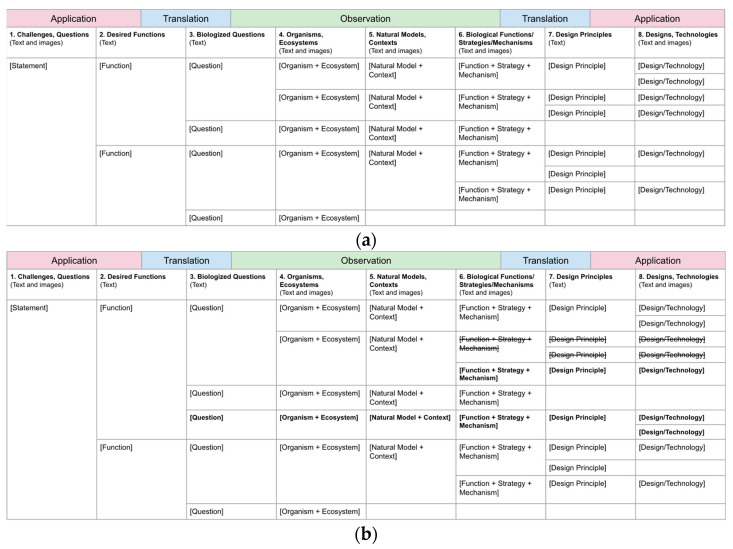
(**a**) An example of a tracking table showing variation in a hypothetical project progression involving a challenge statement, two desired functions, and four biologized questions with corresponding organisms and their ecosystems. Three natural models are carried forward, resulting in five function, strategy, and mechanisms sets leading to six design principles and five ideas for designs or technologies. (**b**) The same project after iterations resulting in new variations (in bold) with others abandoned (in bold with strikethrough).

The second tool is a process tracking table for organizing the output generated in each of the steps during a biomimicry process that places the steps in sequence (columns) and captures the alternatives generated for each (rows), as shown in Figure 5a. All of the steps in the process have the potential for variations to be generated, and it is possible to iterate on some or all of the steps to refine the outcomes, as shown in Figure 5b. As a result, there can be many outputs to track. We have found the table to be particularly useful for managing a biomimicry design project, including all of the variations and iterations explored.

## 4. Results

### 4.1. Using Primary Literature to Map Design Insights

**Figure 6:**
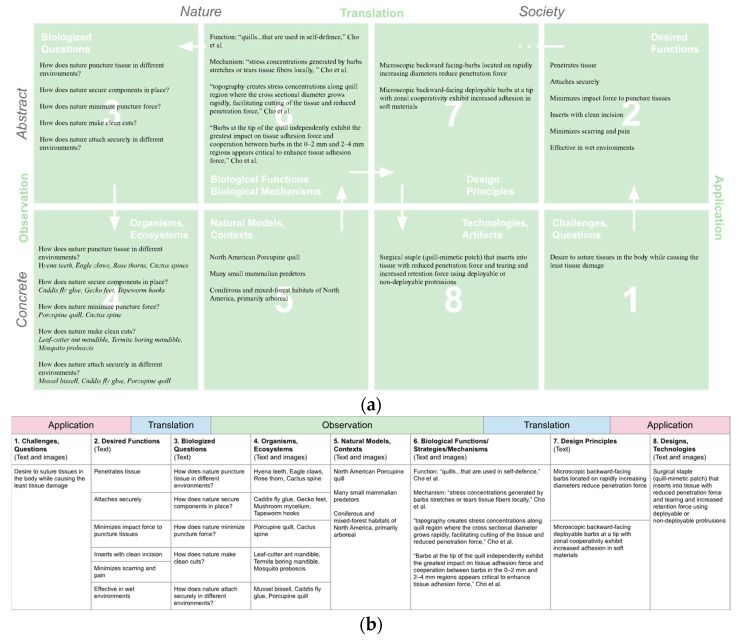
(**a**) Application of the process template using primary literature to create a worked example that references research described by Cho et al. [31] on the properties of the barbed quills of the North American Porcupine and application of insights for a surgical staple, USPTO Pat. US20130331792A1 [32]. Research articles are referenced and mapped onto the framework to illustrate design moves for students involving Observation, Translation, and Application skills. (**b**) The mapped example in a corresponding process tracking table.

In our classroom pedagogy, we illustrate and explore the biomimicry framework by guiding students through a synchronous exercise that involves mapping insights from primary literature to the framework’s design moves. The activity begins with a close reading of a selected scientific paper, such as the Cho et al. [31] example shown in Figure 6. Together, we walk through the paper’s experiments and results, analyzing the data in detail. Students then identify and articulate the function, mechanism, and design insights presented in the research and populate the corresponding sections of the framework table. This activity is followed by a class-wide discussion that deepens understanding and enables students to make connections across the stages of the biomimicry design process.

### 4.2. Applying the Design Process Tools

**Figure 7:**
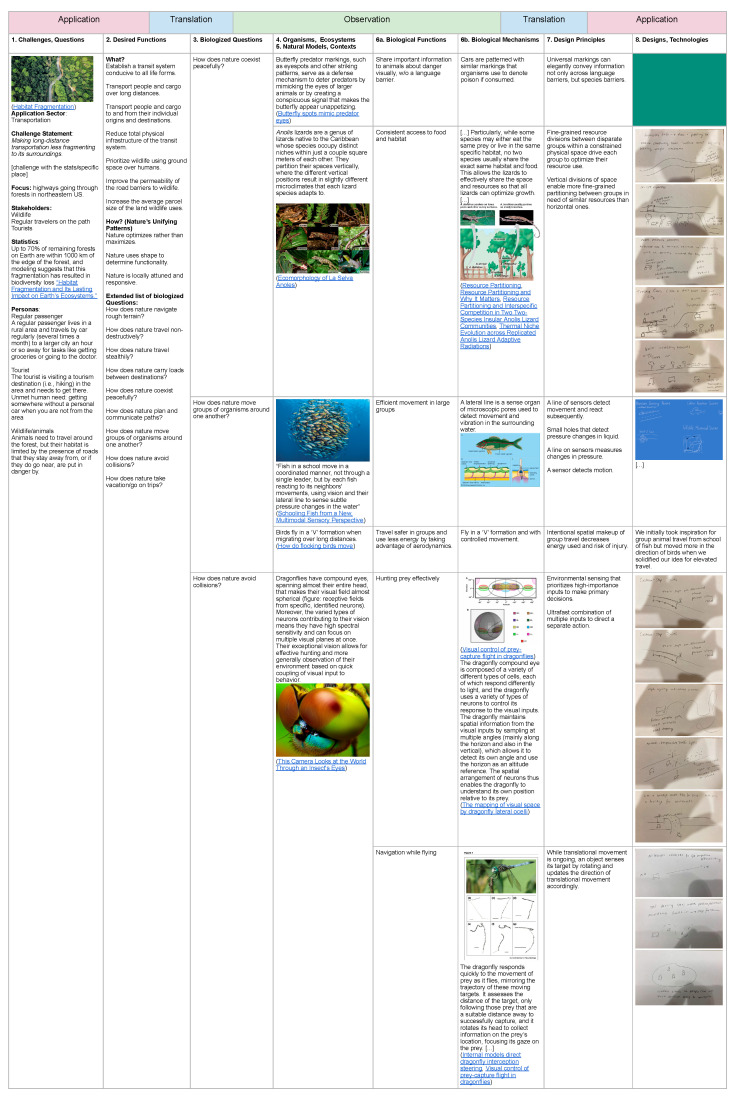
Example of student work with the process tracking table. The assignment is a complete ATOTA process on the student-chosen challenge of minimizing the impact of transportation to environmental surroundings. Steps 4 and 5, identifying organisms and ecosystems and natural models and contexts, were combined in the same column, and step 6, identification and study of biological functions and mechanisms, was split into two columns. The material presented is a portion of what the students produced.

Engineering students used the tracking table to guide and manage complete biomimicry design projects. Figure 7 illustrates the challenge-to-biology or ATOTA process results from an open-ended team-based student project with 4–5 students per team in an upper-level biomimicry course. Selected rows of the completed tracking table (Figure 7) show student work through the steps of the process. Below, we provide a narrative of the project prompt to illustrate how the framework and tools supported a biomimicry design process.

The ATOTA project served as a culminating project following a series of shorter and intermediate length (OTA) design exercises and skill-building activities in Observation, Translation, and Application that took place throughout the semester [3]. The culminating project asked student teams to integrate all aspects of the framework into a complete challenge-to-biology-to-design (ATOTA) process. Students selected open-ended challenges within broad themes such as urban planning, transportation, or disaster relief, and applied the framework and tools to carry out a full biomimicry design process. The project unified student understanding of individual design moves and emphasized iteration, systems thinking, and primary-party awareness in a relevant design context.

Beginning in the Application stage, students defined the challenge they selected, in this case, reducing the environmental fragmentation and impacts caused by transportation, and researched contextual information including statistics characterizing the challenge. They articulated relevant functions using structure–function (SF) and conditions-conducive-to-life (CCL) criteria and then developed a set of biologized challenge questions based on these functions. They then selected the questions that guided their exploration of the natural world, and during the Observation stage, they identified and researched natural models aligned with the corresponding functions. Students identified natural models, researched biological mechanisms, and translated these mechanisms into design principles to gain relevant insights. These insights informed initial application concepts, which were later refined and evaluated against primary-party needs and functional criteria during the final Application stages.

The tracking table was intended to serve as both a scaffold and a visual management tool for navigating the biomimicry design process. We observed that the tool supported iterative development, allowing students to revisit and refine earlier stages as their understanding deepened. Feedback was provided both at each stage and holistically. The structure of the table made it easy to identify, track, and adjust lines of inquiry as the project progressed. The application of an elevated road with traffic organized in clusters to minimize disruption was arrived at after evaluation of the alignment of project goals and primary-party-defined metrics.

## 5. Discussion

### 5.1. Primary Literature Mapping as an Early Exercise

The guided reading of primary literature, paired with mapping insights onto the biomimicry framework table, served as an effective exercise to underscore the importance of understanding natural model mechanisms in depth. This process concretized the design moves of biomimicry and enhanced students’ critical thinking, engagement with scientific texts, and their understanding of natural systems at the mechanistic level. Using primary literature to populate the framework allowed students to trace and articulate the design process clearly, making connections across stages and solidifying their learning with a structured reference that they could revisit during open-ended design projects. The exercise also emphasized the value of detailed, mechanistic analysis with scale and context in the biomimicry process. The study and referencing of the North American porcupine quill in this example (Figure 6) highlighted how the scale and structure of its barbs are essential for translating insights into applications such as the surgical staple that was a resulting application of the knowledge. By reading and mapping primary literature together in class, students were able to appreciate how observation, research, and in-depth analysis are carried through in biomimetic design.

### 5.2. Student Work with the Design Process Tools

The biomimicry process template and tracking table facilitate design work by enabling discussion, exploration, and collaboration with peers and instructors throughout the process. In one example team project, students undertook the challenge of minimizing ecological fragmentation and impact caused by transportation systems (Figure 7). They began by exploring biologized challenge questions focused on terrain-crossing strategies, drawing inspiration from organisms such as goats, worms, ants, and other natural models with distinct mechanisms for navigating complex landscapes. The table’s visual format, enhanced with images provided by the student designers, facilitated the synthesis and discussion of ideas and facilitated the revision and redirection of focus areas pursued as needed. As the project progressed, students broadened their focus areas to include natural models for collision avoidance, such as the behaviors of dragonflies and schooling fish. The table allowed them to visualize and revisit areas of the design process at a glance, revealing patterns in their research and opportunities for revision. The students then expanded their work to consider biologized challenge questions related to resource and niche utilization, deriving insights from anole lizards known for microhabitat specialization that reduces competition and enables species coexistence within trees. The layered and iterative lines of inquiry, taking into consideration SF and CCL criteria, enabled exploration of a range of strategies, which, when paired with primary-party-informed evaluation of application ideas, facilitated a complete biomimicry design process, including sustainability considerations [3].

The tracking table supported ongoing project management, iterative refinement and collaboration across all stages of the process. Its structured layout made it easier to discuss progress and directions with students, provide targeted feedback, and monitor the progression of ideas. Across all student project teams, project directions shifted as new insights emerged from research and reflection. The tracking table played a critical role in managing these transitions, making visible the natural models, functions, and design alternative mappings under consideration, supporting decision making about which directions to pursue or revise. In the sample work provided, students initially focused on environmentally low-impact transportation. They were encouraged to expand their idea space, which resulted in richer, more integrated application concepts with niche differentiation and approaches to partitioning space for coexistence. The table served as a reference, allowing students to track their work over time, identify areas for improvement, and reflect on which stages required more development. Features like strikethroughs and notes were used to mark the less productive paths and highlight future directions, helping students to engage with the iterative nature of the biomimicry process. Some students appeared to stick with early design choices, e.g., natural models selected, even when they were given feedback to change, which could be evidence of design fixation [13,14] or the sunk-cost fallacy at work due to the effort put in filling out a tracking table.

## 6. Conclusions

We engage with challenges in biomimicry education and practice, offering actionable methods for navigating complexity and supporting interdisciplinary collaboration. The novel conceptual framework developed gives us a common language for talking about different design moves and sets of moves to support student work and discussion about the biomimicry design process. The framework is differentiated by its emphasis on primary biomimicry design moves and their relationship within a single design cycle through abstraction and analogy, as well as deemphasizing the problem-solving perspective. Our approach aligns with prior work emphasizing abstraction techniques and the need for clear mappings between biological and technological domains [2,11].

The process template scaffolds learning and understanding the process, making the steps and associated skills involved explicit in a form that facilitates their practice and understanding. The tracking table scaffolds the biomimetic process and enables critical design behaviors such as variation, reflection, debugging, and iteration throughout, supporting structured and flexible engagement with inspiration from biology. We have demonstrated application in an educational context, showing how students used the framework and tools to complete full challenge-to-biology-to-design processes, adapt project directions, and refine ideas through iterative practice. The framework has been combined with the curricular scaffolding described previously [3] to provide a clear biomimicry process that addresses SF and CCL considerations and makes the biomimicry design process accessible and navigable.

## Data Availability

Data associated with this study are available upon request.

## Abbreviations

The following abbreviations are used in this manuscript:

A	Application
BID	Biologically Inspired Design
CCL	Conditions Conducive to Life
O	Observation
SF	Structure Function
T	Translation
